# Quantifying circulating hypoxia-induced RNA transcripts in maternal blood to determine *in utero* fetal hypoxic status

**DOI:** 10.1186/1741-7015-11-256

**Published:** 2013-12-09

**Authors:** Clare Whitehead, Wan Tinn Teh, Susan P Walker, Cheryl Leung, Sonali Mendis, Luke Larmour, Stephen Tong

**Affiliations:** 1 Translational Obstetrics Group, Department of Obstetrics and Gynaecology, University of Melbourne, Mercy Hospital for Women, Heidelberg 3084 VIC, Australia; 2 Monash Health, Monash Medical Centre, Clayton 3168, VIC, Australia; 3 Perinatal Research Centre, Department of Obstetrics and Gynaecology, University of Melbourne, Mercy Hospital for Women, Heidelberg 3084, VIC, Australia

**Keywords:** Circulating mRNA, Pregnancy, Fetal growth restriction, Fetal hypoxia, Biomarker, Diagnostics

## Abstract

**Background:**

Hypoxia *in utero* can lead to stillbirth and severe perinatal injury. While current prenatal tests can identify fetuses that are hypoxic, none can determine the severity of hypoxia/acidemia. We hypothesized a hypoxic/acidemic fetus would up-regulate and release hypoxia-induced mRNA from the fetoplacental unit into the maternal circulation, where they can be sampled and quantified. Furthermore, we hypothesized the abundance of hypoxia induced mRNA in the maternal circulation would correlate with severity of fetal hypoxia/acidemia *in utero*. We therefore examined whether abundance of hypoxia-induced mRNA in the maternal circulation correlates with the degree of fetal hypoxia *in utero*.

**Methods:**

We performed a prospective study of two cohorts: 1) longitudinal study of pregnant women undergoing an induction of labor (labor induces acute fetal hypoxia) and 2) pregnancies complicated by severe preterm growth restriction (chronic fetal hypoxia). For each cohort, we correlated hypoxia induced mRNA in the maternal blood with degree of fetal hypoxia during its final moments *in utero*, evidenced by umbilical artery pH or lactate levels obtained at birth. Gestational tissues and maternal bloods were sampled and mRNAs quantified by microarray and RT-PCR.

**Results:**

Hypoxia-induced mRNAs in maternal blood rose across labor, an event that induces acute fetal hypoxia. They exhibited a precipitous increase across the second stage of labor, a particularly hypoxic event. Importantly, a hypoxia gene score (sum of the relative expression of four hypoxia-induced genes) strongly correlated with fetal acidemia at birth. Hypoxia-induced mRNAs were also increased in the blood of women carrying severely growth restricted preterm fetuses, a condition of chronic fetal hypoxia. The hypoxia gene score correlated with the severity of ultrasound Doppler velocimetry abnormalities in fetal vessels. Importantly, the hypoxia gene score (derived from mRNA abundance in maternal blood) was significantly correlated with the degree of fetal acidemia at birth in this growth restriction cohort.

**Conclusions:**

Abundance of mRNAs coding hypoxia-induced genes circulating in maternal blood strongly correlates with degree of fetal hypoxia/acidemia. Measuring hypoxia-induced mRNA in maternal blood may form the basis of a novel non-invasive test to clinically determine the degree of fetal hypoxia/acidemia while *in utero*.

## Background

Significant fetal hypoxia causing injury or death can occur acutely, such as during labor [1], or it can occur chronically as a result of poor placental function (placental insufficiency). Chronic hypoxia arising from placental insufficiency can also cause severe fetal growth restriction (FGR).

When FGR occurs at significantly preterm gestations, the risks of stillbirth are high and clinicians must judge the optimal time to deliver the fetus. They are required to balance the probability of stillbirth, neonatal death or permanent disability (caused by severe fetal acidemia) if the pregnancy is left to continue versus the risks of iatrogenic prematurity if the preterm fetus is delivered unnecessarily early in gestation [2]. To help make these decisions, tests of fetal well-being are used to determine the likelihood that the fetus is significantly hypoxic. These include the cardiotocograph (reports fetal heart rate patterns) [3], biophysical profile (reports the presence/absence of fetal movement, breathing, tone and amniotic fluid volume on ultrasound) [4] and Doppler waveform velocimetry analysis of fetal vessels [5]. While access to these tests has improved perinatal outcomes, FGR fetuses are still lost to stillbirth or neonatal demise or they suffer significant perinatal injury [6]. In a large study of 604 live-born cases of preterm FGR (<33 weeks gestation), major morbidity occurred in 35.9% of cases and mortality was 21.5% [7]. As such, there is scope for significant improvements in clinical outcomes.

A potential limitation of existing tests is that they observe fetal physiological responses to hypoxia [8]. As such, significant heterogeneity may be expected, where the threshold of hypoxia/acidemia required to provoke specific physiological responses captured by these tests will vary between fetuses. In addition, current tests only provide a likelihood that significant hypoxia is likely to be present. Importantly, none are validated to provide a quantitative estimate of the fetal blood pH/lactate concentrations (fetal acidemia). An approach measuring fetal hypoxia at a biochemical/molecular level may be a more direct strategy to determine the degree of fetal hypoxia/acidemia *in utero*.

The discovery that fetoplacentally derived mRNA is constantly released into the maternal blood from early pregnancy until delivery raises the possibility of a new way to monitor for the presence of fetal hypoxia/acidemia [9,10]. We hypothesized a hypoxic fetus would up-regulate and release hypoxia-induced mRNA into maternal blood. Furthermore, the relative abundance of such transcripts may quantitatively correlate with the degree of fetal acidemia. Thus, measuring hypoxia-induced mRNA abundance in the maternal circulation might form the basis of a non-invasive test to estimate *in utero* fetal blood pH concentrations. We therefore investigated whether hypoxia-induced mRNA abundance in maternal blood correlated with the degree of fetal hypoxia/acidemia *in utero*.

## Methods

### Study participants and specimens

Participants were recruited from two tertiary hospitals in Melbourne (Monash Medical Centre and Mercy Hospital for Women). We obtained approval from The Mercy Hospital for Women Human Research Ethics Committee (MHW R10/02) and The Southern Health Research Ethics Committee B (MMC 09154B). All women provided written informed consent.

To examine acute hypoxia, a prospective study was undertaken in the birth suite. Maternal whole blood was collected from women undergoing induction of labor at term. An intravenous cannula was inserted at recruitment reserved for sample collection for the study. Serial blood samples were collected: prior to induction and commencement of uterine contractions; at commencement of the second stage of labor (full dilatation) and at delivery. Fetal umbilical artery blood samples and placental biopsies were collected immediately after delivery. Fetal hypoxic/acidemic status was determined by measuring umbilical artery blood lactate levels. Thirty women with documented fetal umbilical artery blood lactate levels at delivery and complete sampling were matched according to gestation, parity and maternal characteristics to identify a normoxic (fetal umbilical cord lactate <6 mmol/L) and hypoxic cohort (fetal umbilical lactate >6 mmol/L).

To examine chronic hypoxia, serial maternal whole blood samples were collected from 20 women carrying severely growth restricted preterm fetuses and 30 controls. Severe FGR was defined as a customized birthweight <10^th^ centile (http://www.gestation.net, Australian parameters) requiring iatrogenic delivery prior to 34 weeks’ gestation with uteroplacental insufficiency (asymmetrical growth + abnormal umbilical artery Doppler velocimetry +/- oligo-hydramnios). Women with superimposed preeclampsia were included. Control blood samples (n = 30) were collected from women carrying an appropriately grown fetus (matched for gestation, parity and maternal characteristics) and subsequently delivered at term without complications.

Preterm placental samples (n = 8) were collected from women delivering preterm an appropriate grown fetus without hypertensive disease or histological evidence of chorioamnionitis. We only included those in the FGR cohort who delivered by caesarean to avoid the potential bias caused by acute hypoxia of labor. For the FGR cohort, fetal hypoxic status at delivery was determined by measuring fetal blood pH levels obtained from the umbilical artery at birth.

### Sample collection

A total of 2.5 mls of either maternal peripheral whole blood and/or fetal umbilical cord blood samples were collected in PAXgene whole blood RNA tubes (PreAnalytix, Hombrechtikon, Switzerland). According to the manufacturer’s instructions, they were stored at room temperature for 24 hours, then at -80C until processing.

Placental biopsies were obtained immediately after delivery from the maternal side of the placenta avoiding the decidua and fetal membranes (placental samples from the FGR cohort were all after caesarean section). Biopsies were washed in sterile phosphate buffered saline, snap frozen and stored at -80°C until processing.

### RNA preparation

Total RNA was extracted using the Paxgene miRNA system (PreAnalytix/BD) according to the manufacturer’s instructions. Total RNA was extracted from placental tissue using the mirVana isolation kit (Ambion, Austin, TX, USA) according to the manufacturer’s instructions. Genomic DNA was removed using DNAse treatment. RNA concentration and purity was measured using a NanoDrop ND100 spectrophotometer (Thermo Scientific, Pittsburgh, PA, USA). Microarray sample quality were evaluated further by the BioAnalyser 2100 system (Agilent, Santa Clara, CA, USA).

### Microarray analyses

RNA samples were hybridized to Illumina Human Ref-8 Expression Beadchips for the labor ward study and the Illumina HumanHT-12 Expression Beadchips (Illumina Inc., San Diego, CA, USA) for the FGR study. Beadchip processing was performed by the Australian Genome Research Facility (Melbourne, VIC, Australia) according to the manufacturer’s instructions. Scanned images were analyzed using Illumina GenomeStudio. Gene expression analysis was performed using BioConductor in R (http://www.r-project.org) after quality control, preprocessing, background subtraction and normalization was performed. Linear modeling was performed using the Limma package (http://www.bioinf.wehi.edu.au/limma/) and fold change calculated using the t-test adjusted for multiple comparisons using the Benjamini and Hochberg methodology for false discovery rate. Unsupervised hierarchical clustering and principal component analysis were performed to illustrate how well the patient groups could be separated on the basis of the different molecular signatures. GSEA software (http://www.broadinstitute.org/gsea) was used to investigate over-representation of biological pathways, comparing published biological pathways and the gene-set developed in this study.

Microarray data are available in the ArrayExpress database (http://www.ebi.ac.uk/arrayexpress) under accession number E-MTAB-2054.

### Validation with quantitative real-time reverse-transcription polymerase chain reaction analysis

Real-time reverse-transcription polymerase chain reaction (RT-PCR) validation of the 48 gene molecular signature was performed using the Taqman Hypoxia TLDA (Applied Biosystems, Carlsbad, CA, USA) and specific Taqman primers and probes for the candidate four gene signature (Applied Biosystems). Reverse transcription of 200 ng of total RNA was performed using Superscript Vilo or Superscript III (Invitrogen, Carlsbad, CA, USA). All RT-PCRs were performed in triplicate using multiple negative controls including RT and no-template controls. We chose the combined expression of *Gapdh*, *B2m* and *Gusb* as our internal control having validated that their expression was not altered by hypoxia in these samples. The comparative CT method was used to determine relative expression. Non-parametric statistical tests were used for comparison of gene expression and the Bonferroni correction for multiple comparisons was used where appropriate.

## Results

### Expression of hypoxia-induced mRNA in fetal blood and placenta sampled from fetuses acutely hypoxic during labor

During labor, each uterine contraction abrogates maternal blood flow within the myometrium, decreasing placental oxygenation [1]. Fetuses are rendered progressively hypoxic as labor advances [1]. Therefore, labor is effectively an *in vivo* functional ‘model’ of acute human fetal hypoxia.

We first examined whether hypoxia-induced mRNA transcripts are increased in gestational tissues (fetal blood and placenta) in the presence of acute fetal hypoxia caused by labor. Blood lactate concentrations in the umbilical artery (from the placenta) at birth are measured by clinicians to retrospectively determine whether the fetus was genuinely hypoxic during its final moments *in utero*, where levels >6 mmol/L are considered elevated [11]. Therefore, we grouped our cohort according to whether umbilical artery lactate concentrations were high (>6 mmol/L; hypoxia cohort) or not (<6 mmol/L; controls).

**Figure 1:**
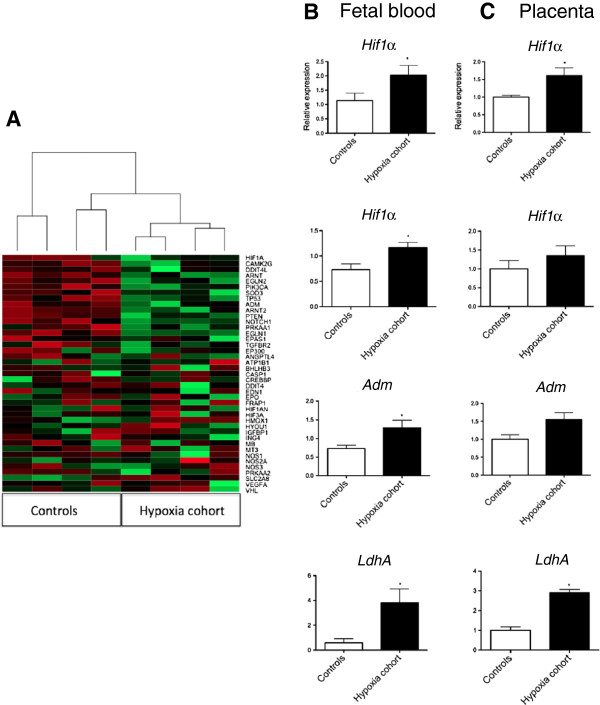
**Expression of hypoxia-induced mRNA in gestational tissues obtained after labour. (A)** Cluster analysis and a heatmap of 41 hypoxia induced genes in blood of fetuses from the hypoxia cohort (where umbilical artery lactate concentrations sampled from the placenta soon after birth were >6.0 mmol/L) or controls (umbilical artery lactate <6.0 mmol/L). Green indicates increased expression, red is decreased expression. Expression of *hypoxia inducible factor* (*Hif*)*1α*, *Hif2α, adrenomedullin* (*Adm*) and *lactate dehydrogenase A* ((*LdhA*) in **(B)** fetal blood and **(C)** placenta obtained from the hypoxia cohort (n = 4) or controls (n = 4). Mean and S.E.M. are graphed. *P <0.05.

Microarray and geneset enrichment analysis on mRNA in fetal blood obtained from the umbilical artery at birth revealed significant over-representation of hypoxia-associated pathways in the hypoxia cohort. There was global up-regulation of 41 hypoxia-induced transcripts selected from the microarray in the hypoxia cohort compared to controls (Figure 1A). PCR confirmed up-regulation of four hypoxia-induced transcripts in the fetal blood among the hypoxia cohort compared to controls: *hypoxia inducible factor 1α* (*Hif1α*), *Hif2α*, *adrenomedullin* (*Adm*) and *lactate dehydrogenase A* (*LdhA*; Figure 1B). These genes were also up-regulated in placentas from the hypoxia cohort compared to controls (Figure 1C). Thus, hypoxia-induced mRNAs increase in gestational tissues with fetal hypoxia.

### Expression of hypoxia-induced mRNA in maternal blood sampled at the moment of delivery in cases where the fetus was significantly hypoxic at birth

Next, we investigated whether hypoxia-induced transcripts were increased in maternal blood sampled at the moment of birth, comparing the hypoxic cohort (umbilical artery lactate concentrations >6 mmol/L) with controls. We performed a prospective labor ward study, recruiting women undergoing an induction of labor. We placed a second intravenous cannula reserved for collecting samples for the study (Additional file 1: Table S1 lists clinical details). The ‘moment of birth’ sample was taken when the head was on view at the perineum (vaginal opening) and delivery was imminent.

**Figure 2:**
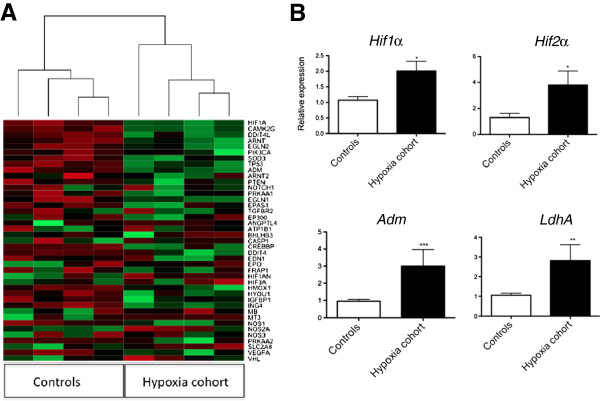
**Expression of hypoxia-induced mRNA in maternal blood from women in labor. (A)** Cluster analysis and heatmap of 41 hypoxia induced transcripts in maternal blood sampled moments before delivery from the hypoxia cohort (umbilical artery lactate concentrations sampled at birth were >6.0 mmol/L) or controls (umbilical artery lactate <6.0 mmol/L). Green indicates increased expression, red is decreased expression. **(B) ***Hypoxia inducible factor* (*Hif*)*1α*, *Hif2α*, *adrenomedullin* (*Adm*) and *lactate dehydrogenase A* (*LdhA*) expression in maternal blood sampled moments before birth from the hypoxia cohort (n = 14) or controls (n = 16). Mean and S.E.M. are graphed. *P <0.05. **P <0.01. ***P <0.001.

Genomewide microarray and geneset enrichment analysis of mRNA isolated from maternal blood samples taken at the moment of birth showed over-representation of hypoxia pathways in the hypoxia cohort (not shown). Expression of 41 hypoxia-induced mRNA selected from the array were globally increased in the hypoxia cohort (Figure 2A). Samples grouped according to fetal hypoxic status when unsupervised hierarchical clustering was performed on the 41 genes subset. PCR confirmed *Hif1α*, *Hif2α*, *Adm* and *LdhA* were up-regulated in the hypoxia cohort (Figure 2B). Therefore, trends seen in maternal blood (Figure 2A, B) mirrored those seen in gestational tissues (Figure 1A-C).

### Expression of hypoxia-induced mRNA in maternal blood sampled longitudinally across labor

**Figure 3:**
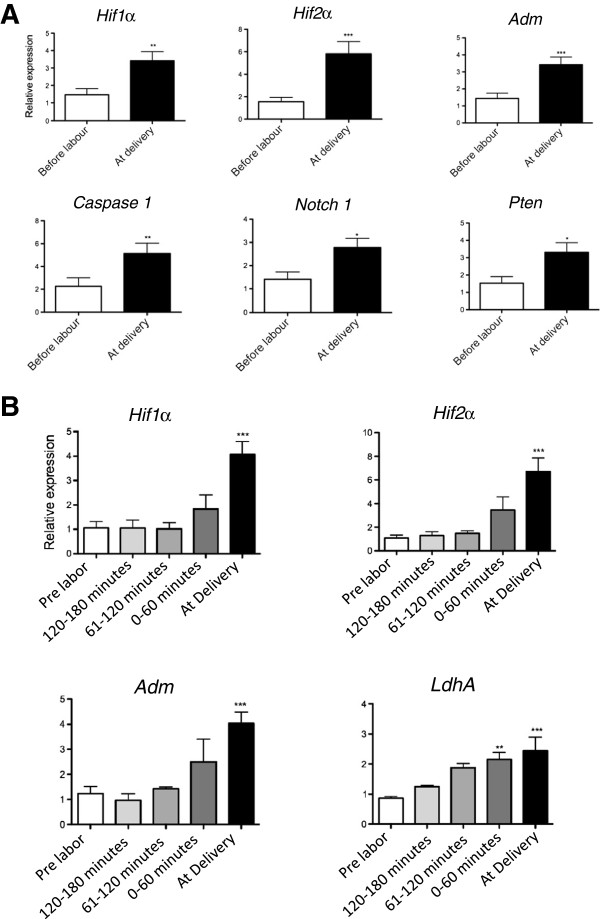
**Expression of hypoxia-induced mRNA in maternal blood from women sampled across labor. (A) ** Expression of six hypoxia-induced transcripts in paired maternal blood sampled before labor was commenced and moments before delivery. Data were generated using a Taqman PCR array. **(B) ***Hypoxia inducible factor* (*Hif*)*1α*, *HIF2α*, *adrenomedullin* (*Adm*) and *lactate dehydrogenase A* (*LdhA*) expression in serial samples taken before labor was commenced (white bars; n = 30), during the second stage (full cervical dilatation) at either 120 to 180, 61 to 120 or 0 to 60 minutes prior to delivery (gray bars, sampling from the cohort of 30 were split among the three bars), and at the moment of birth (black bar; n = 30). Thus, white and gray bars depict mRNA expression of genes in maternal blood at time-points straddling the beginning and end of the first stage of labor (mean (± SD) 486 (± 242) minutes between sample collection), while the gray and black bars straddle the second stage of labor (mean (± SD) 44 (± 55) minutes between sample collection). Data were generated using Taqman PCR. Mean and S.E.M. are graphed. *P <0.05 **P <0.01 ***P <0.001.

We next investigated whether hypoxia-induced transcripts in maternal blood acutely increase within hours of new onset acute fetal hypoxia, by examining whether hypoxia-induced mRNA in maternal blood increase across labor. A total of 22 of 44 hypoxia-induced transcripts measured on a PCR array were significantly up-regulated in maternal blood sampled at the moment of vaginal birth compared to paired samples obtained prior to the onset of labor (see Additional file 1: Table S2; Figure 3A graphs six genes from the array). A further eight genes on the array trended towards an increase (≥1.3-fold increase).

To exclude the possibility of a non-specific global rise in mRNA transcripts in maternal blood across labor, we assessed five transcripts coding growth-related genes by PCR. None of these significantly increased across labor (Additional file 1: Figure S1).

The second stage of labor (full cervical dilatation until birth) is shorter in duration than the first stage (from onset of contractions until full cervical dilatation), but particularly hypoxic for fetuses where rapid decreases in fetal arterial pO2, base excess and pH occur [1,12-14]. We measured *Hif1α*, *Hif2α*, *Adm* and *LdhA* expression in maternal blood obtained from samples straddling the first stage and second stage of labor (mean (± SD) 486 (± 242) minutes between sample collection) and compared the relative increase to paired samples straddling the second stage of labor (mean (± SD) 44 (± 55) minutes between sample collection). There were only minimal increases in gene expression across the first stage of labor but far steeper increases across the second stage (Figure 3B). Thus, hypoxia-induced transcripts do not gradually increase across labor linearly with time (this might be expected to occur if these transcripts were released primarily in response to general inflammation that occurs during labor [15] rather than fetal hypoxia). Instead, they rose far more steeply during the much shorter period of the second stage of labor. We suggest the likely explanation is that fetuses are more hypoxic during the second stage [1,12-14].

### Correlation between hypoxia-induced mRNA in maternal blood sampled at the moment of birth with fetal hypoxic status at birth

**Figure 4:**
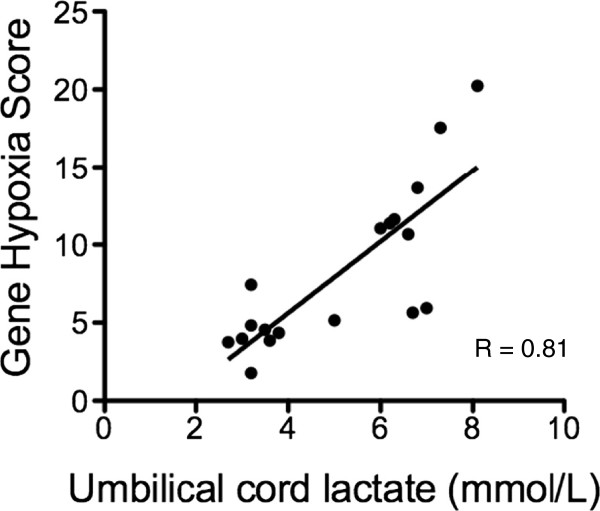
**Correlation between a gene hypoxia score and umbilical artery lactate concentrations sampled after labour.** Gene hypoxia score is the sum of relative *hypoxia inducible factor * (*Hif*)*1α*, *Hif2α*, *adrenomedullin* (*Adm*) and *lactate dehydrogenase A* (*LdhA*) expression in maternal blood taken at the moment of delivery. *P* <0.0001.

We next examined whether hypoxia-induced transcripts in maternal blood correlate with the degree of fetal hypoxia/acidemia *in utero*. We developed a hypoxia gene expression score by summing relative expression of *Hif1α*, *Hif2α*, *Adm* and *LdhA* in maternal blood obtained at the moment of birth and correlating the scores with umbilical artery lactate concentrations measured at delivery (lactate concentrations in the umbilical artery positively correlate with degree of *in utero* fetal acidemia around the time of birth). There was a highly significant correlation between the hypoxia gene expression score and fetal blood lactate concentrations at delivery (Figure 4; r = 0.81, *P* <0.0001). We conclude abundance of hypoxia-induced transcripts in maternal blood correlates with the fetal hypoxia/acidemia *in utero* in the setting of acute hypoxia caused by labor.

### Expression of hypoxia-induced mRNA in maternal blood sampled from women with severe preterm growth restricted fetuses

**Figure 5:**
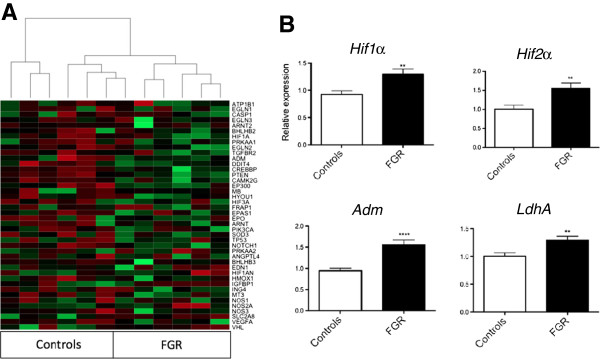
**Expression of hypoxia-induced mRNA in maternal blood from cases of severe fetal growth restriction (FGR). (A)** Heatmap of 42 hypoxia induced genes in maternal blood sampled from controls and FGR cohort. Green indicates increased expression, red is decreased expression. **(B) ***Hypoxia inducible factor* (*HIF*)*1α*, *Hif2α*, *adrenomedullin* (*Adm*) and *lactate dehydrogenase A* (*LdhA*) expression in maternal blood sampled from controls (n = 30) and FGR cohort (n = 20). Mean and S.E.M. are graphed. **P <0.01 ****P <0.0001.

We next examined severe preterm fetal growth restriction (FGR; n = 20, Table 1 shows the clinical details), a disease of chronic *in utero* hypoxia [8,16]. Controls (n = 30) were uncomplicated pregnancies where maternal blood was obtained at the same gestational age as the FGR cohort. Microarray and gene enrichment analysis on mRNA isolated from maternal blood demonstrated an over-representation of hypoxia pathways in the FGR cohort compared to controls. Samples clustered according to whether fetuses were growth restricted or not. There was global up-regulation of hypoxia-induced mRNA in the FGR cohort (Figure 5A). PCR confirmed *Hif1α*, *Hif2α*, *Adm* and *LdhA* were significantly up-regulated in the FGR cohort compared to controls (Figure 5B). They were also up-regulated in placentas obtained from FGR cases compared to two sets of controls: gestation matched preterm placentas not complicated by FGR and placentas obtained from healthy pregnancies delivering at full term (Additional file 1: Figure S2).

**Table 1:** Patient characteristics for fetal growth restriction and control cohorts

		**FGR (n = 20)**	**Control (n = 30)**	***P *** **=**
Maternal age (yrs)^a^		30.4 (5.9)	29.5 (.8)	0.65
Parity (% primiparous)		66	66	1.00
Gestational age at delivery (weeks)^a^		29 + 5 (21)	40 + 1 (10)	<0.0001
Gestational age at sampling (weeks)^a^		29 + 5 (21)	29 + 0 (18)	0.33
Mode of birth (%)	Vaginal	0	70	<0.0001
	Instrumental	0	10	
	Caesarean section	100	20	
Birthweight (gms)^a^		885 (273)	3,403 (244)	<0.0001
Birthweight centile^a^		3 (3)	45 (16)	<0.0001
Five-minute Apgar score^a^		7 (1)	9 (1)	<0.001
Neonatal admission (%)		100	7	<0.0001
Perinatal death (%)		13	0	0.10
Smoking (%)		13	0	0.10
Gestational hypertension/pre-eclampsia (%)		40	0	<0.001
Pre-gestational diabetes (%)		6	0	0.03
Umbilical artery Doppler USS waveforms (%)	REDF^b^	30	n/a	
	AEDF^c^	40		
	SDR^d^	30		

FGR, fetal growth restriction; ^a^Mean (SD), ^b^Reversed End Diastolic Flow, ^c^Absent End Diastolic Flow, ^d^(Raised) Systolic/Diastolic Ratio.

### Expression of hypoxia induced mRNA in maternal blood in the FGR cohort, split according to whether preeclampsia was also present

To examine the possibility that co-existent preeclampsia may affect expression of hypoxia-induced mRNA in maternal blood (and thus, be a confounder), we split our FGR cohort according to whether there was concurrent preeclampsia (n = 8) or not (n = 12). mRNA expression of *Hif1α*, *Hif2α*, *Adm* and *LdhA* were all significantly elevated in both FGR cohorts (that is, FGR with concurrent preeclampsia, and FGR without preeclampsia; see Additional file 1: Figure S3) compared to gestationally-matched controls (healthy pregnancies that progressed to delivery at term of an infant with a normal birth weight). Importantly, mRNA expression levels of all four genes were no different between the FGR cohort with concurrent preeclampsia and FGR without preeclampsia. These data suggest mRNA coding hypoxia induced genes are increased in the presence of severe FGR, irrespective of the presence of preeclampsia.

### Correlation between abundance of hypoxia-induced RNA in maternal blood with severity of Doppler velocimetry abnormalities

**Figure 6:**
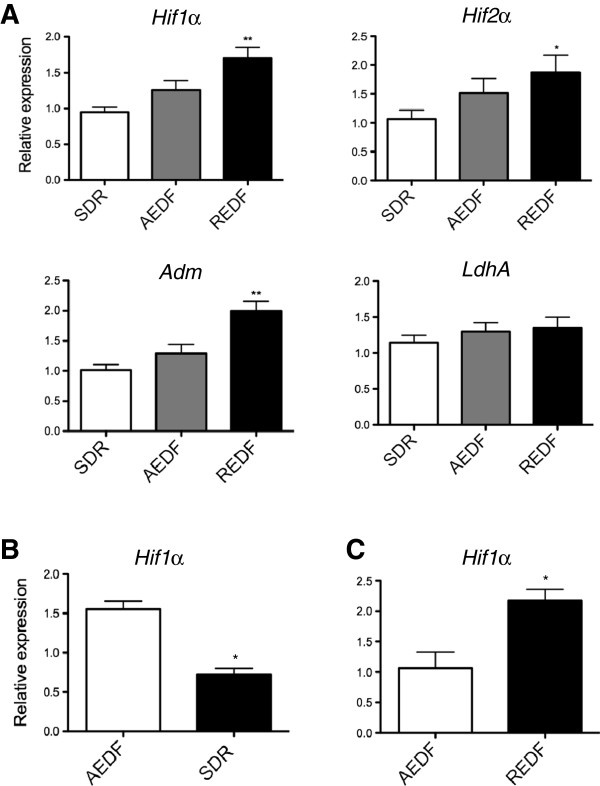
**Expression of hypoxia-induced mRNA in maternal blood in association with different umbilical artery velocimetry waveforms. (A)***Hypoxia inducible factor* (*HIF*)*1α*, *Hif2α*, *adrenomedullin* (*Adm*) and lactate dehydrogenase A (LDHA) expression in maternal blood sampled from women in the FGR cohort, grouped according to umbilical artery velocimetry waveform findings (SDR = Raised systolic/diastolic ratio; AEDF = Absent end diastolic flow; REDF = Reversed end diastolic flow). AEDF and REDF results were compared with SDR findings. **(B)** Expression of *Hif1α* in paired samples of maternal blood obtained around 24 hours apart from the FGR cohort, where umbilical artery velocimetry findings either acutely improved **(C)** or worsened after administration of corticosteroids. Samples obtained at the time of the ultrasound examinations and corticosteroids were given between the ultrasound examinations. Mean and S.E.M. are graphed. *P <0.05, **P <0.01.

Ultrasound Doppler velocimetry of blood flow in the umbilical artery is used clinically to serially monitor hypoxic status of growth-restricted fetuses *in utero*[8]. A raised systolic-diastolic ratio (SDR) represents the mildest abnormality, absent end diastolic flow (AEDF) is associated with an increased likelihood of significant fetal hypoxia [17] while reversed end diastolic flow (REDF) has the strongest association with acidemia and imminent fetal death. We measured *Hif1α*, *Hif2α*, *Adm* and *LdhA* expression in maternal blood samples taken in parallel with umbilical artery Doppler assessment and confirmed a progressive increase with worsening Doppler findings (Figure 6A).

### Correlation between dynamic changes in hypoxia-induced mRNA in maternal circulation and acute changes in umbilical artery Doppler waveforms

Corticosteroids are often administered via intramuscular injection to the mother to accelerate fetal lung maturation, in preparation for preterm birth [18,19]. They can induce acute, but transient improvements of the umbilical artery Doppler waveforms over 24 to 72 hours (for example, from AEDF to raised SDR) [20], while in some cases, waveforms worsen [21]. Paired maternal blood samples were taken just prior to, and 24 hours after, corticosteroid administration. Decreased expression of *Hif1α* (Figure 6B) was observed when the Doppler waveforms improved and conversely, increased expression of *Hif1α* (Figure 6C) was observed when waveforms deteriorated after corticosteroid administration. This suggests circulating hypoxia-induced transcripts in maternal blood promptly change in parallel with very acute alterations in presumed fetal hypoxic status.

### Correlation between hypoxia-induced mRNA in maternal blood sampled on the day of delivery with acidemic status of FGR infants at the moment of birth

**Figure 7:**
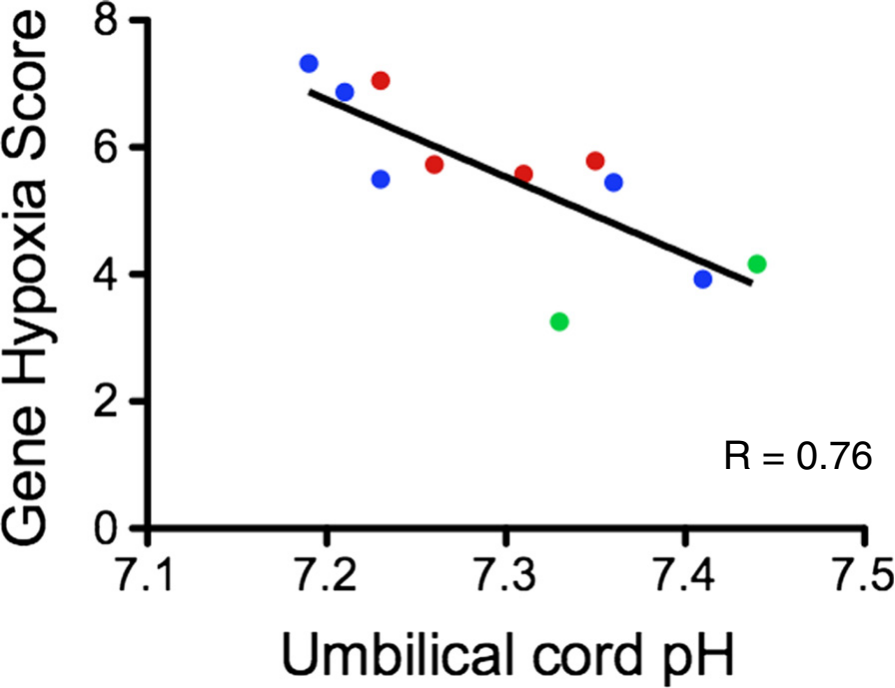
**Correlation between a gene hypoxia score and umbilical artery pH levels sampled at birth.** Gene hypoxia score is the sum of relative hypoxia inducible factor (*Hif*)*1α*, *Hif2α*, *adrenomedullin* (*Adm*) and *lactate dehydrogenase A* (*LdhA*) expression in maternal blood on the day of delivery. Samples were taken from the fetal growth restriction cohort. *P* = 0.008. Colors indicate umbilical artery waveform findings seen on the day of delivery: Blue = Raised systolic/diastolic ratio, Green = Absent end diastolic flow, Red = Reversed end diastolic flow.

Lastly, we correlated the hypoxia gene expression score (sum of the relative expression of *Hif1α*, *Hif2α*, *Adm* and *LdhA*) in maternal blood from samples taken on the day of delivery with fetal acidemic status at birth (pH of fetal blood drawn from the umbilical artery) in the FGR cohort. We observed a strong correlation (Figure 7; r = 0.76, *P* = 0.008) between the hypoxia gene score and umbilical artery pH levels at birth (that is, fetal acidemic status, where decreasing pH correlates with increasing fetal acidemia). In contrast, there did not appear to be an obvious relationship between different severities of Doppler waveform velocimetry findings observed on the day of delivery and final fetal acidemic status (Figure 7). We conclude the hypoxia gene expression score correlates with fetal acidemic status *in utero* in FGR.

## Discussion

Significant fetal acidemia at birth is strongly associated with perinatal death and adverse perinatal complications, including permanent neurological disability [6] and cerebral palsy [22]. In a study of 60 preterm fetuses delivered at ≤28 weeks gestation, an umbilical artery blood pH of ≤7.15 was strongly associated with severe adverse neurological outcomes (sensitivity 30% at 98% specificity) compared with higher pH levels [6]. In another study of 604 neonates delivered at ≤33 weeks gestation, an umbilical cord pH of ≤7.20 was associated with a 4.2 likelihood ratio of fetal death [7]. Therefore, a non-invasive test that can estimate fetal acidemic status could help clinicians’ better time delivery. While current non-invasive antenatal tests can identify fetuses at higher risk of being acidemic, none have been validated to accurately estimate the degree of fetal acidemia *in utero*.

Here we have presented evidence to suggest quantifying hypoxia-induced mRNA in the maternal circulation may be a novel approach to determining *in-utero* fetal hypoxic status. Hypoxia-induced transcripts in the maternal circulation appear tightly correlated with expression in human gestational tissues, and they dynamically change with acute alterations in presumed fetal hypoxic status. Furthermore, we generated a hypoxia gene expression score that sums the relative abundance of mRNA in the maternal circulation that code four hypoxia-induced genes. This score appeared to be highly correlated with acute (labor cohort) and chronic (FGR cohort) fetal hypoxia.

While the measurement of free mRNA in the maternal circulation has been studied previously, we believe our study represents a significant conceptual advance. Previous studies have proposed the use of free mRNA as a ‘static’ tool, where levels are measured once in order to either diagnose [23,24] or predict pregnancy complications [25-27]. Here we propose serial measurements to observe dynamic changes within the same patient, monitoring hypoxic status over time and delivering when significant acidemia is predicted.

The cardiotocograph is the mainstay of monitoring to identify hypoxia during labor. While it performs well in identifying the presence of fetal hypoxia (85% sensitivity) [1], its specificity is notoriously poor because heart rate decelerations, including late decelerations, can either be caused by hypoxia or be induced by mechanical reflex autonomic responses unrelated to hypoxia. As a result, use of the cardiotocograph results in unnecessary interventions [28]. Ours may be the first ‘theoretical’ non-invasive test for women in labor that can determine the degree of *in utero* fetal acidemia. The speed of current PCR technologies means such a test is not feasible as a clinical tool to make decisions during labor but improvements in nucleic acid detection technologies might make such a test possible in the future.

We have also presented evidence suggesting hypoxia-induced mRNA in the maternal circulation correlates with acidemic status of FGR fetuses’ *in utero*. It is conceivable that day-to-day clinical decisions regarding timing of an FGR fetus can await the results of a PCR result performed using machines available today. Therefore, our test may have a role in situations where current tests of fetal well-being are equivocal and the clinician is left unsure whether the fetus should be delivered. This occurs quite frequently. A prospective study examining a preterm FGR cohort found biophysical profile results were discordant with the umbilical artery Doppler findings in 55% of cases [29]. Thus, a test that can provide a reliable estimate of *in utero* fetal blood pH levels in such situations may help clinicians decide whether immediate delivery is warranted.

A limitation of our study is that we have not decisively proven the hypoxia-induced mRNA we are measuring in the maternal blood originates from the fetoplacental unit. This may be possible with the use of next-generation sequencing technologies where sequence information could be used to identify the origin of mRNA transcripts (maternal or fetal). However, we have presented strong circumstantial evidence to suggest the hypoxia induced mRNA are of fetoplacental origin: 1) they increase with situations of likely severe acute and chronic fetal hypoxia, 2) they correlate with an increase of hypoxic mRNA transcripts in gestational tissues, and 3) their relative abundance displays a highly significant and tight correlation with fetal acidemic status at birth. Ultimately, if hypoxia-induced transcripts in maternal blood were validated to reflect fetal acidemic status, it would not be absolutely essential to establish their origin, although a fetoplacental source seems the most likely.

To translate our potential test to the monitoring of fetuses with severe FGR, our test requires validation with a study of larger numbers. Such a validation study could also help determine whether clinical factors, such as smoking and maternal obesity, alter hypoxia induced mRNA levels in maternal blood. We are currently undertaking such a large prospective validation study.

Furthermore, in this proof of concept study, we summed the relative expression of mRNA in maternal blood that codes *Hif1α*, *Hif2α*, *LdhA* and *Adm* to generate a gene hypoxia score. These genes were chosen on the basis of their biology; the former three have central roles in the hypoxic response [30], and *Adm* is both hypoxic regulated [31] and very highly expressed in placenta. Future studies should bioinformatically screen other hypoxia-induced genes to develop the most accurate test to determine degree of *in utero* fetal acidemia. Finally, it may be more optimal to develop a clinical test that expresses mRNA abundance by copy number rather than relative expression.

In conclusion, we have presented evidence to show measuring circulating hypoxia-induced transcripts in maternal blood may be a promising approach to clinically assess fetal hypoxic status *in utero*. It may be useful to help clinicians’ time delivery, especially in cases of severe preterm FGR, potentially improving perinatal outcomes and decreasing rates of stillbirth.

## Conclusions

Abundance of mRNAs coding hypoxia-induced genes circulating in maternal blood strongly correlates with the degree of fetal hypoxia/acidemia, and they dynamically change with acute alterations in presumed fetal hypoxic status. Furthermore, a hypoxia gene expression score that sums the relative abundance of mRNAs in the maternal circulation was highly correlated with acute (labor ward cohort) and chronic (FGR cohort) fetal hypoxia/acidemia. Therefore, measuring hypoxia-induced mRNA in maternal blood may form the basis of a novel non-invasive test to clinically determine the degree of fetal hypoxia/acidemia while *in utero*.

## Abbreviations

Adm: *Adrenomedullin*; AEDF: Absent end diastolic flow; FGR: Fetal growth restriction; Hif1α: *Hypoxia inducible factor 1α*; Hif2α: *Hypoxia inducible factor 2α*; LdhA: *Lactate dehydrogenase A*; mRNA: Message Ribonucleic acid; PCR: Polymerase chain reaction; PET: Pre-eclampsia; REDF: Reversed end diastolic flow; SDR: Raised systolic-diastolic ratio.

## Competing interests

The authors declare that they have no competing interests.

## Authors’ contributions

ST conceived the study. ST, CW and SW designed the study. CW, WT, CL, SM and LL collected samples. CW performed the laboratory work. CW analyzed the data, including the microarray bioinformatic analysis. ST, CW and SW wrote the first draft of the paper and all authors provided input and approved the final manuscript.

## Pre-publication history

The pre-publication history for this paper can be accessed here:

http://www.biomedcentral.com/1741-7015/11/256/prepub

## Supplementary Material

Additional file 1: Figure S1 mRNA of growth genes in maternal blood across labor. Relative expression of mRNA coding five growth genes in paired maternal blood obtained just before labour commenced and at moment of delivery. *GH*: *Growth Hormone*; *Adam12*: *Disintegrin and metalloproteinase domain-containing protein 12*; *IGFR1*: *Insulin Growth Factor 1 receptor*; *IGF: Insulin Growth Factor. ***Figure S2.** Expression of hypoxia-induced mRNA in placentas complicated by severe preterm fetal growth restriction. Expression of five hypoxia induced mRNA in placental specimens obtained from cases of preterm controls (no fetal growth restriction, n = 8), term controls (delivered >37 weeks gestation, n = 8) and severe preterm fetal growth restriction (FGR, delivered <34 weeks gestation, n = 20). Comparisons were made between controls and the FGR cohort. *Hif: hypoxia inducible factor*; *Adm: adrenomedullin*; *LdhA: lactate dehydrogenase A*. *P < 0.05, **P < 0.01, ***P < 0.001. **Figure S3.** mRNA in maternal blood among the FGR cohort, grouped according to whether pre-eclampsia was present. Comparisons between fetal growth restriction (FGR) with no pre-eclampsia (FGR - no PET; n = 12) versus FGR with co-existent pre-eclampsia (FGR + PET; n = 8) were non-significant for all four genes (P ≥ 0.41). Remaining comparisons, as shown in the graphs, were made between both FGR groups and gestationally matched controls (controls were pregnancies that progressed to delivery of an infant of normal birthweight at term). *Hif: hypoxia inducible factor*; *Adm: adrenomedullin*; *LdhA: lactate dehydrogenase A*. *P < 0.05, **P < 0.01, ***P < 0.001, ****P < 0.0001. **Table S1.** Clinical characteristics for the cohort who laboured. The group was stratified according to whether the fetus was hypoxic (umbilical artery lactate concentrations >6.0 mmol/L) or not (umbilical artery lactate concentrations <6.0 mmol/L) at birth. **Table S2.** Expression of hypoxia-induced mRNAs in maternal blood before labor compared to moment of birth. Click here for file
