# Supplementary material for: Quantifying circulating hypoxia-induced RNA transcripts in maternal blood to determine in utero fetal hypoxic status
Source: BMC Med. 2013 Dec 9;11:256. doi: 10.1186/1741-7015-11-256 (PMC4029381; doi:10.1186/1741-7015-11-256)
Supplement: Additional file 1: Figure S1 — mRNA of growth genes in maternal blood across labor. Relative expression of mRNA coding five growth genes in paired maternal blood obtained just before labour commenced and at moment of delivery. GH: Growth Hormone; Adam12: Disintegrin and metalloproteinase domain-containing protein 12; IGFR1: Insulin Growth Factor 1 receptor; IGF: Insulin Growth Factor. Figure S2. Expression of hypoxia-induced mRNA in placentas complicated by severe preterm fetal growth restriction. Expression of five hypoxia induced mRNA in placental specimens obtained from cases of preterm controls (no fetal growth restriction, n = 8), term controls (delivered >37 weeks gestation, n = 8) and severe preterm fetal growth restriction (FGR, delivered <34 weeks gestation, n = 20). Comparisons were made between controls and the FGR cohort. Hif: hypoxia inducible factor; Adm: adrenomedullin; LdhA: lactate dehydrogenase A. *P < 0.05, **P < 0.01, ***P < 0.001. Figure S3. mRNA in maternal blood among the FGR cohort, grouped according to whether pre-eclampsia was present. Comparisons between fetal growth restriction (FGR) with no pre-eclampsia (FGR - no PET; n = 12) versus FGR with co-existent pre-eclampsia (FGR + PET; n = 8) were non-significant for all four genes (P ≥ 0.41). Remaining comparisons, as shown in the graphs, were made between both FGR groups and gestationally matched controls (controls were pregnancies that progressed to delivery of an infant of normal birthweight at term). Hif: hypoxia inducible factor; Adm: adrenomedullin; LdhA: lactate dehydrogenase A. *P < 0.05, **P < 0.01, ***P < 0.001, ****P < 0.0001. Table S1. Clinical characteristics for the cohort who laboured. The group was stratified according to whether the fetus was hypoxic (umbilical artery lactate concentrations >6.0 mmol/L) or not (umbilical artery lactate concentrations <6.0 mmol/L) at birth. Table S2. Expression of hypoxia-induced mRNAs in maternal blood before labor compared to moment of birth. [file 1741-7015-11-256-S1.pdf]

Supplementary Materials

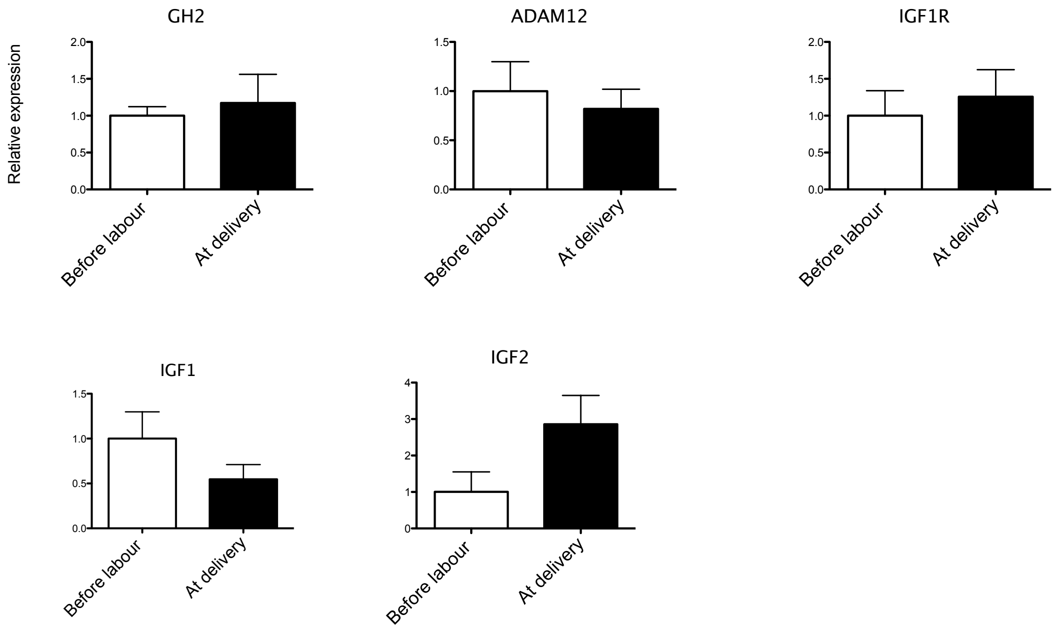

Figure S1: Expression of growth genes across labour.

Relative expression of mRNA coding five growth genes in paired maternal blood obtained just before labour commenced and at moment of delivery. GH: Growth Hormone; ADAM12: Disintegrin and metalloproteinase domain-containing protein 12; IGFR 1: Insulin Growth Factor 1 receptor.

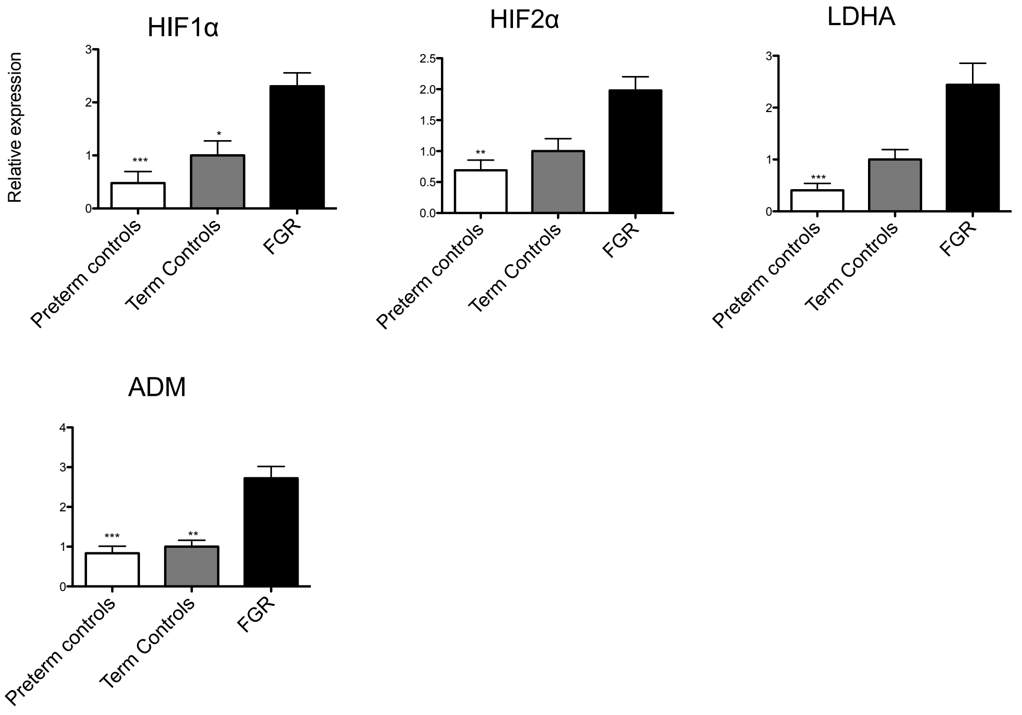

Figure S2: Expression of hypoxia-induced mRNA in placentas complicated by severe preterm FGR.

Relative mRNA expression of five hypoxia induced mRNA in placental specimens obtained from preterm controls (n=8), term controls (delivered >37 weeks gestation, n=8) and from cases of severe preterm fetal growth restriction (FGR, delivered under 34 weeks gestation, n=20). Comparisons were made between controls and the FGR cohort. * $P<0.05$, ** $P<0.01$, *** $P<0.001$.

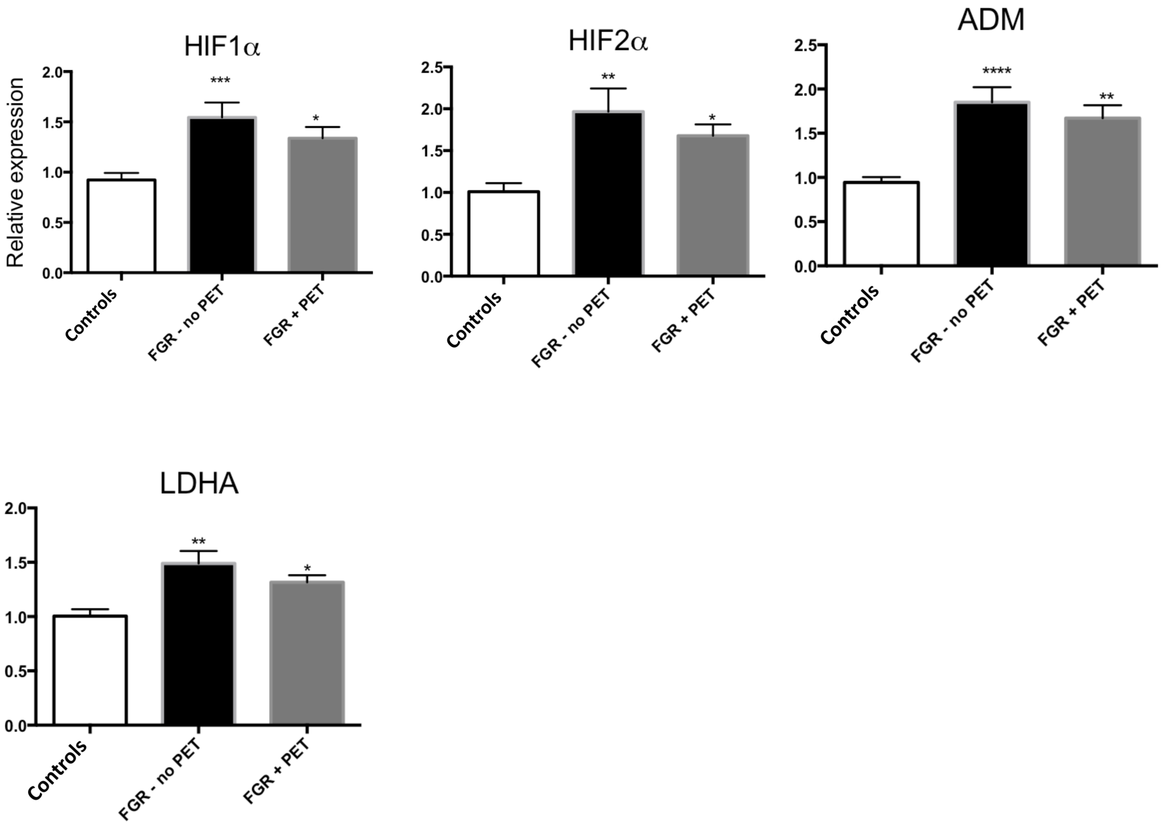

Figure S3: Expression of hypoxia-induced mRNA blood, with the FGR cohort split according to whether there was concurrent preeclampsia (FGR + PET, n=8) or not (FGR no PET n=12).

Comparisons between FGR - no PET vs FGR + PET were non-significant for all four genes (P≥0.41). Remaining comparisons, as shown in the graphs, were made between both FGR groups and gestationally matched controls (pregnancies that progressed to delivery of an infant of normal birthweight at term). *P<0.05, ** P<0.01, *** P<0.001, **** P<0.0001

Table S1: Clinical characteristics for the cohort who were in labour

The group was stratified according to whether the fetus was hypoxic (umbilical artery lactate concentrations >6.0 mmol/L) or not (umbilical artery lactate concentrations <6.0 mmol/L) at birth.

		High lactate (n=14)	Low lactate (n=16)	P=
Age (yrs) [a]		28.3 (6)	29.7 (5)	0.54
Parity (% Primiparous)		79	75	1.00
Gestational Age (weeks) [a]		39+4 (9)	40 (13)	0.40
Length of 1st stage (mins) [a]		414 (204)	540 (2620	0.18
Length of 2nd stage (mins) [a]		40 (49)	49 (61)	0.69
Delivery Mode (%)	SVD	64	31	0.27
	Instrumental	21	38	
	Caesarean	14	31	
Birthweight (gms) [a]		3055 (523)	3385 (580)	0.114
1 minute apgar [b]		9 (8-9)	9 (7-9)	0.94
5 minute apgar [b]		9 (9-9)	9 (9-9)	0.75
Cord lactate (mmol/L) [a]		6.6 (1)	3.7 (1)	<0.0001
Admission to neonatal unit (%)		36	37.5	1.00

[a]Mean (SD), [b] median (IQR).

Table S2: Changes in expression of Hypoxia-induced mRNAs in paired maternal blood obtained just before labour commenced and samples obtained at the moment of birth.

Gene	Fold change*	p value
**ADM**	**2.4**	**<0.001**
**ANGPTL4**	**2.4**	**<0.001**
**ARNT**	**1.9**	**0.05**
ARNT2	3.5 down	0.11
ATP1B1	1.0	0.92
**BHLHB30**	**1.8**	0.02
CAM2KG	1.5	0.18
**CASP1**	**2.3**	**<0.01**
CREBBP	2.0	0.06
**DDIT4**	**4.2**	**<0.01**
DDIT4L	0.95	0.94
EDN1	1.1	0.45
**EGLN1**	**2.2**	**<0.01**
**EGLN2**	**2.1**	**<0.01**
EGLN3	Low expression	
EPO	Low expression	
**EP300**	**2.8**	**<0.01**
FRAP1	1.5	0.27
**HIF1**	**2.3**	**<0.001**
**HIF1AN**	**2.3**	**<0.001**
**HIF2A**	**3.8**	**<0.001**
HIF3A	Low expression	
**HIG2**	**2.8**	**0.03**
**HMOX1**	**1.7**	**0.03**
**HYOU1**	**1.4**	**0.04**
**ING4**	**1.9**	**<0.01**
IGFBP1	Low expression	
**LDHA**	**2.1**	**<0.06**
MB	Low expression	
MT3	Low expression	
NOS1	Low expression	
NOS2A	Low expression	
NOS3	0.9	0.87
**NOTCH1**	**2.0**	**0.02**
**PIK3CA**	**1.7**	**0.03**
PRKAA1	1.3	0.25
PRKAA2	Low expression	
**PTEN**	**2.2**	**0.01**
SLC2A8	1.4	0.20
**SOD3**	**1.8**	**0.05**
**TGFBR2**	**1.8**	**0.04**
TP53	1.3	0.70
VEGFA	1.5	0.10
VHL	1.5	0.24

* Fold changed measured on a Taqman PCR array.
